# The Effect of Lifestyle Coaching Design on Patient Engagement and Weight Loss in Non-diabetic Patients of a Semaglutide-Supported Digital Obesity Program in the UK: A Comparative Retrospective Cohort Study

**DOI:** 10.7759/cureus.74321

**Published:** 2024-11-23

**Authors:** Louis A Talay, Matt Vickers, Leif Lagesen, Nicole Liu

**Affiliations:** 1 Research, Eucalyptus, Sydney, AUS; 2 Arts and Social Sciences, University of Sydney, Sydney, AUS; 3 Clinical Research, Eucalyptus, Sydney, AUS; 4 Nutrition, Eucalyptus, Sydney, AUS; 5 Product Development, Eucalyptus, London, GBR

**Keywords:** digital health, lifestyle intervention, multidisciplinary health care, obesity care, patient engagement levels, semaglutide

## Abstract

Digital weight-loss services (DWLSs) that supplement continuous lifestyle coaching with semaglutide therapy have shown promise in delivering continuous and effective obesity care. However, the extent to which lifestyle coaching design influences patient engagement and program effectiveness is unknown. This study retrospectively analysed several engagement markers and weight loss percentage over 16 weeks in a large semaglutide-supported DWLS in the UK (n=154). The comparative analysis found that patients who received lifestyle coaching that was proactive and personalised sent a statistically higher number of messages to their health coach (Mean=19.37 vs Mean=8.55) and opened the program app more frequently (Mean = 49.31 days vs Mean = 40.06 days) than patients whose coaching was reactive and standardised. Mean 16-week weight loss was 10.1% in the proactive group compared to 8.9% in the reactive group, but the difference was not statistically significant. A two-sample t-test found that female patients (Mean = 9.76%) tended to lose more weight than male patients (Mean = 6.88%), (*t*(152) = 1.89, *p *= 0.04). The findings add vital layers of nuance to the emerging literature on semaglutide-supported DWLSs, indicating that a proactive, personalised coaching approach leads to better patient engagement, but that such engagement is not correlated with better short-term weight-loss or program adherence outcomes. Moreover, the cohort’s comparably higher mean weight loss relative to previous real-world semaglutide studies lend support to the advice of leading global health institutions of using the medication only as an adjunct to multidisciplinary lifestyle therapy. Investigators should expand on this research by conducting comparable studies over a longer period and with medication-only control groups.

## Introduction

Obesity is defined by the World Health Organization as "a chronic complex disease defined by excessive fat deposits that can impair health" [1]. Specifically, the WHO considers adults with a body mass index (BMI) of 30kg/m^2^ or more to be living with obesity. The global prevalence of obesity has surged more than threefold since the 1970s, with around three billion people now living with excess weight or obesity [1]. Many experts attribute this rise to the multifaceted nature of the disease, linking it to various economic, social, environmental, and neurological factors [2-4]. This complexity has made it difficult for weight-loss interventions to produce consistent and long-term success for most individuals [5,6]. Moreover, the accessibility and adherence to effective weight-loss programs may be a crucial yet underappreciated factor in the global rise in obesity [7].

In recent years, glucagon-like peptide-1 receptor agonists (GLP-1 RAs) have emerged as a promising category of weight-loss drugs. Multiple clinical trials have highlighted their unprecedented effectiveness in both diabetic and non-diabetic groups [8-10]. In non-diabetic groups who combine semaglutide therapy with lifestyle counselling, participants average a reduction between 14.9 and 16 percent of baseline weight after 68 weeks [9,11]. Arguably more significantly, over 85 percent of patients in these cohorts lost at least 5 percent of their baseline weight, the figure widely regarded as clinically meaningful weight loss [12]. These results are often attributed to the effect of GLP-1 RAs on modulating the neurological pathways involved in satiety [13,14]. However, some commentators have raised concerns that these medications are being framed as a cure-all for obesity, rather than as a supplement to sustained lifestyle changes [15,16]. Leading health organizations like the World Health Organization (WHO) and the National Institute for Health and Care Excellence (NICE) stress that GLP-1 RAs should only ever be prescribed to obese patients as an adjunct to continuous multidisciplinary lifestyle interventions [17,18].

While this recommended treatment approach is sound, accessing and sticking to lifestyle therapy in traditional healthcare settings remains challenging. People with demanding work or family schedules often find it difficult to attend regular consultations with multidisciplinary teams (MDTs), an issue exacerbated by longer waiting times for GP appointments in countries like the UK [7,19]. Furthermore, many individuals with overweight or obesity face stigma about their condition and are uncomfortable discussing it in person [7,20]. Some have also reported that their GPs fail to offer comprehensive lifestyle advice or referrals to specialists [7]. Finally, many people with overweight or obesity live too far from healthcare providers to reasonably access quality obesity care [21,22].

Digital weight-loss services (DWLSs) have emerged in recent times as a potential solution to these barriers [23]. DWLSs can help eliminate the psychological and geographical hurdles to obesity treatment by removing the need for face-to-face (F2F) interactions. They also address time constraints by offering services through digital platforms, with many providing the option for asynchronous consultations, which don’t require real-time interaction [7,24]. In other words, synchronous DWLSs allow patients to consult clinicians at a location of their convenience, while asynchronous DWLS consultations enable patients the freedom to access care at both a location and time of their choice. Some evidence suggests that younger generations prefer the additional privacy that digital consultations afford them, irrespective of whether they consider the disease stigmatized [25]. However, the quality of GLP-1 RA-supported DWLSs varies widely. Some services follow WHO and NICE guidelines by offering GLP-1 RAs as a supplement to continuous MDT-led lifestyle coaching, while others provide little more than access to GLP-1 RA prescriptions without follow-up care, fueling concerns about the over-reliance on medication for weight management [15,26]. These concerns are compounded by a lack of research on GLP-1 RA-supported DWLSs. At present, only a few quantitative studies have been conducted on such services, including analyses of Australian and British cohorts using liraglutide and semaglutide alongside lifestyle coaching [27-30]. While these studies have shown some promising effectiveness and adherence results, several key questions remain unanswered before these DWLS models can be widely adopted. One of the main questions is the extent to which lifestyle coaching design influences engagement and effectiveness outcomes in GLP-1 RA-supported DWLSs. A previous mixed-methods study found that users prefer personalized and proactive coaching styles [31]. However, that investigation did not assess the extent to which lifestyle coaching preferences affected program outcomes.

In line with the paucity of literature on semaglutide-supported DWLSs, research on comparable real-world interventions in F2F settings is also scarce. Our comprehensive literature review across PubMed, Web of Science and Google Scholar found that only two studies of this nature have been published hitherto on non-diabetic cohorts-retrospective reviews of the medical records of 147 patients from the Mayo Clinic Health System (USA) and 43 patients from an endocrine clinic in Athens, respectively [32,33]. The Mayo Clinic study reported a mean three-month weight loss of 6.3% and a mean six-month weight loss of 11.8%, with 44.2% (65/147) of the study’s three-month cohort failing to provide data at six months. The Greek study observed a median weight loss of 6.6% and 13.3% at months 3 and 6 respectively, with a 3-6-month patient exclusion percentage of 42% (18/43). Notably, semaglutide therapy appeared to be combined with minimal lifestyle guidance in both interventions. In the Mayo Clinic study, the methods section did not make any mention of diet or exercise advice and simply stated in the discussion that the observed weight loss outcomes “may have been associated with other interventions (e.g., lifestyle and diet) that are provided by the weight management clinic” [32]. The study of the Athens endocrine clinic gave a much clearer overview of the lifestyle input that its patients received:

“All patients received counselling sessions about nutrition and regular exercise at the time of initiation and every 12 weeks thereafter by an endocrinologist, following the principles of motivational interviewing. It was left to the discretion of each individual whether they sought regular dietician input, participated in a structured physical activity program, or received behavioural/psychological therapy” [33].

However, it is unlikely that this level of lifestyle counselling would be considered consistent with WHO and NICE guidelines on GLP-1 RA obesity therapy [17,18], as it appears more as an adjunct to the medication rather than the other way around. It is feasible that all of the above-discussed barriers to ongoing multidisciplinary care in F2F settings impacted the capacity of the Mayo and Athens clinics to operate such care models.

This study aims to examine the direct effects of proactive versus reactive lifestyle coaching on patient engagement and weight loss in a large, non-subsidized semaglutide-supported DWLS in the UK. It is believed the study’s outcomes will illuminate the importance of adhering to WHO and NICE guidelines on comprehensive obesity care models and the utility of DWLSs in delivering them.

## Materials and methods

This study derived from an internal lifestyle coaching experiment initiated by the Juniper UK DWLS and adopted a retrospective control design to achieve its aims. Patients were unaware of their group allocation, as Juniper UK reasonably assumed that the proactive coaching protocol would not deliver worse outcomes than the standard (reactive) model. Investigators followed the Strengthening the Reporting of Observational Studies in Epidemiology (STROBE) guidelines throughout the entire investigation. The study’s ethics were approved by the Bellberry Limited Human Ethics Committee on 22 November 2023 (No. 2023-05-563-A-1). All study patients consented to the publication of their de-identified data.

Program overview

The Juniper UK DWLS has operated solely through an app-based platform since its inception in 2021. Prospective users fill out an extensive online pre-consultation form, consisting of over 100 questions about their health history. A pharmacist reviews the responses and often requests additional details such as test results, images, and other medical data to assess program eligibility. Decisions are largely based on the Wegovy prescribing information guidelines, which include body mass index (BMI) cut-offs, contraindications like medullary thyroid cancer, and potential interactions with other medications, such as insulin [34].

Eligible individuals, upon paying an initial monthly fee of £189 (increasing to £299 when the highest Wegovy dose of 2.4mg is reached), are assigned an MDT consisting of a pharmacist, a dietitian or nutritionist with university credentials, and a nurse. All interactions between patients and their MDT are stored in an encrypted central database on Metabase (an open-source business intelligence tool) to optimize care continuity. Access to this data is limited to MDTs and the Juniper UK analytics team. Before consenting to the program, patients are forwarded a detailed summary of GLP-1 RA side effects. Each patient is provided with a standardized set of Bluetooth-enabled scales to track their weight.

**Figure 1:**
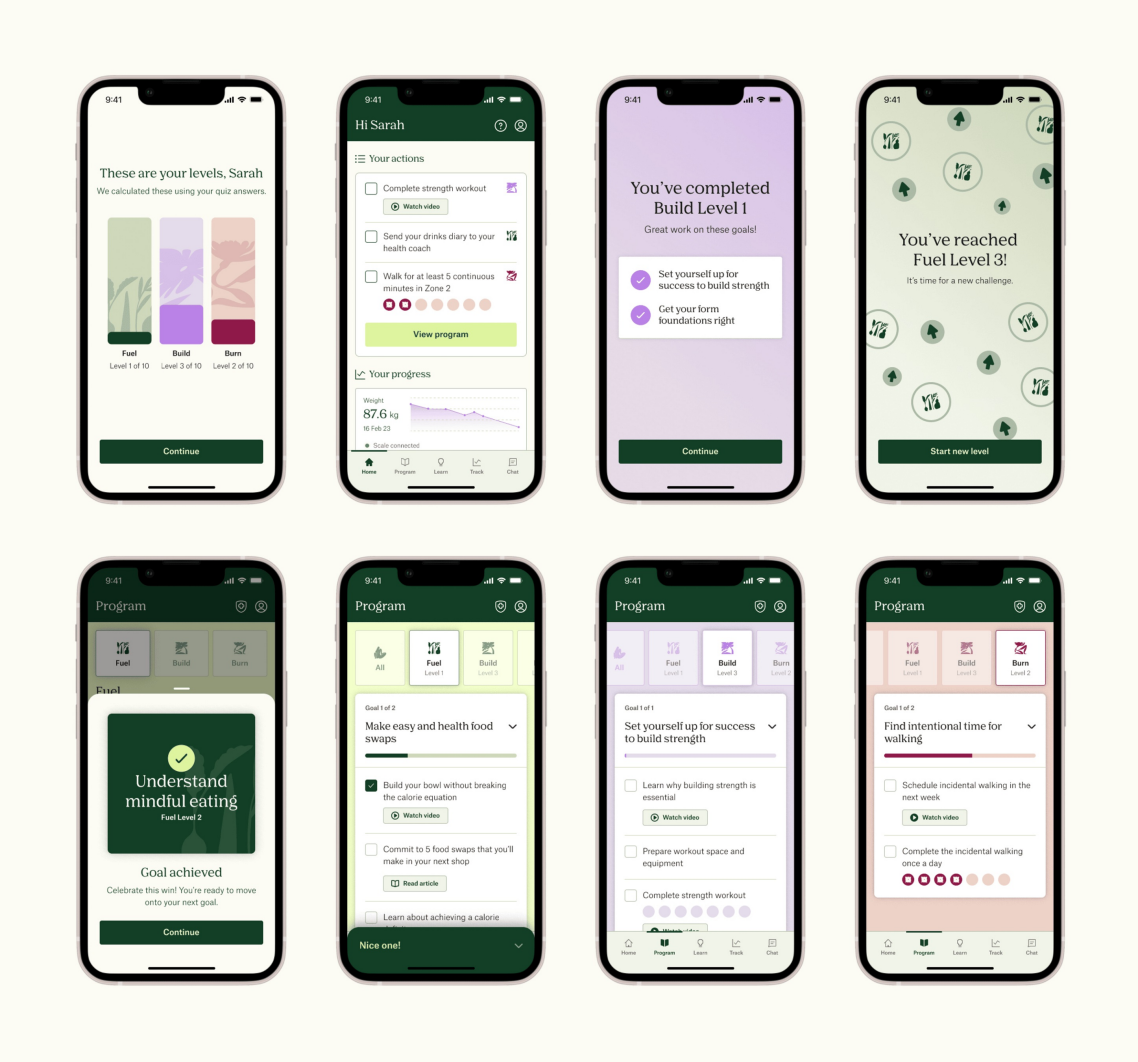
Screen displays of the Juniper app’s “Action” tracker Juniper app: Eucalyptus, Sydney, Australia

Under the standard Juniper UK DWLS, health coaches send patients a short lifestyle quiz to develop diet and exercise plans. The quiz contains questions that assess patient proficiency with resistance and aerobic training and nutritional knowledge. Once the lifestyle plan is sent, patients receive an automated message detailing how to use the Juniper app to optimize their results. Although patients can request changes to their lifestyle plan at any time, they are only prompted to consider adjustments during their first mandatory follow-up appointment at five months (though earlier consultations may be arranged at the clinician's discretion). Similarly, patients can reach out to their health coach whenever they wish, but the app does not proactively prompt them to ask for guidance or update progress using the "Action" or weight tracking features. "Actions" consist of micro diet and exercise goals organized into challenge pillars, which are supported by multimedia educational resources (Figure 1). Patients are sorted into challenge pillars according to their baseline fitness, as indicated in the lifestyle quiz. The only prompt patients receive under the standard program is a biweekly notification to complete a check-in questionnaire, which asks them to enter weight and program satisfaction data and offers them a chance to report adverse events. Completing the check-in is optional, and no further reminders are sent. Therefore, the standard Juniper DWLS provides reactive rather than proactive lifestyle coaching.

In December 2023, the Juniper UK strategy team decided to assess the value of a proactive coaching model. This decision followed an internal review, which found that many patients were not consistently engaging with any of the program app’s functionalities such as the "Actions" feature. To conduct this assessment, they delivered proactive coaching to 100 patients who subscribed to Wegovy-supported treatment from January 12, 2024. Patients were selected on a 1-to-1 basis, with every other patient delivered standard Juniper therapy. The proactive lifestyle coaching protocol included the following differences from the standard "reactive" Juniper program: (i) An introductory program explainer video, with a step-by-step presentation of each app function; (ii) an additional lifestyle preference quiz to provide health coaches with greater capacity for personalizing diet and exercise programs; (iii) automated prompts to update "Actions" every three days; (iv) requirement of setting accountability dates for each goal; (v) two notifications to complete bi-weekly check-in quizzes.

Patients who received proactive coaching followed the same Wegovy titration schedule as standard Juniper UK patients. This schedule adheres to Wegovy prescribing information guidelines and is as follows: 0.25mg weekly for weeks 1 to 4, 0.5mg for weeks 5 to 8, 1mg for weeks 9 to 12, 1.7mg for weeks 13 to 16, and 2.4 mg for week 17 onwards. Due to company budget constraints, the assessment was cancelled after 17 weeks, rather than running for six months as originally planned. 

Participants

The first 200 patients who subscribed to Wegovy-supported Juniper treatment in the UK from January 12 2024 were allocated to either standard or proactive coaching on a 1-to-1 basis. Patient eligibility was determined by a UK-qualified pharmacist, who followed Wegovy prescribing guidelines for weight-loss therapy. These guidelines include the following BMI cutoffs: 27kg/m^2^ or greater (overweight) for any patient of non-Caucasian ethnicity and/or at least one weight-related comorbidity such as sleep apnea or symptomatic cardiovascular disease; or 30kg/m^2^ or greater for anyone else. Exclusion criteria included the following contraindications: a personal or family history of medullary thyroid carcinoma or patients with multiple endocrine neoplasia type 2; acute pancreatitis; a previous acute kidney injury; hypoglycaemia; a severe mental health condition; acute gallbladder disease; known hypersensitivity to semaglutide or any of the product components; and patients with type 1 or type 2 diabetes. Pharmacists used their discretion in determining whether patients on other oral medications could safely take semaglutide without experiencing interactions with the latter’s gastric emptying effect. The sole study-specific inclusion criterion was that patients submitted weight data between 100 and 120 days after program commencement.

**Figure 2:**
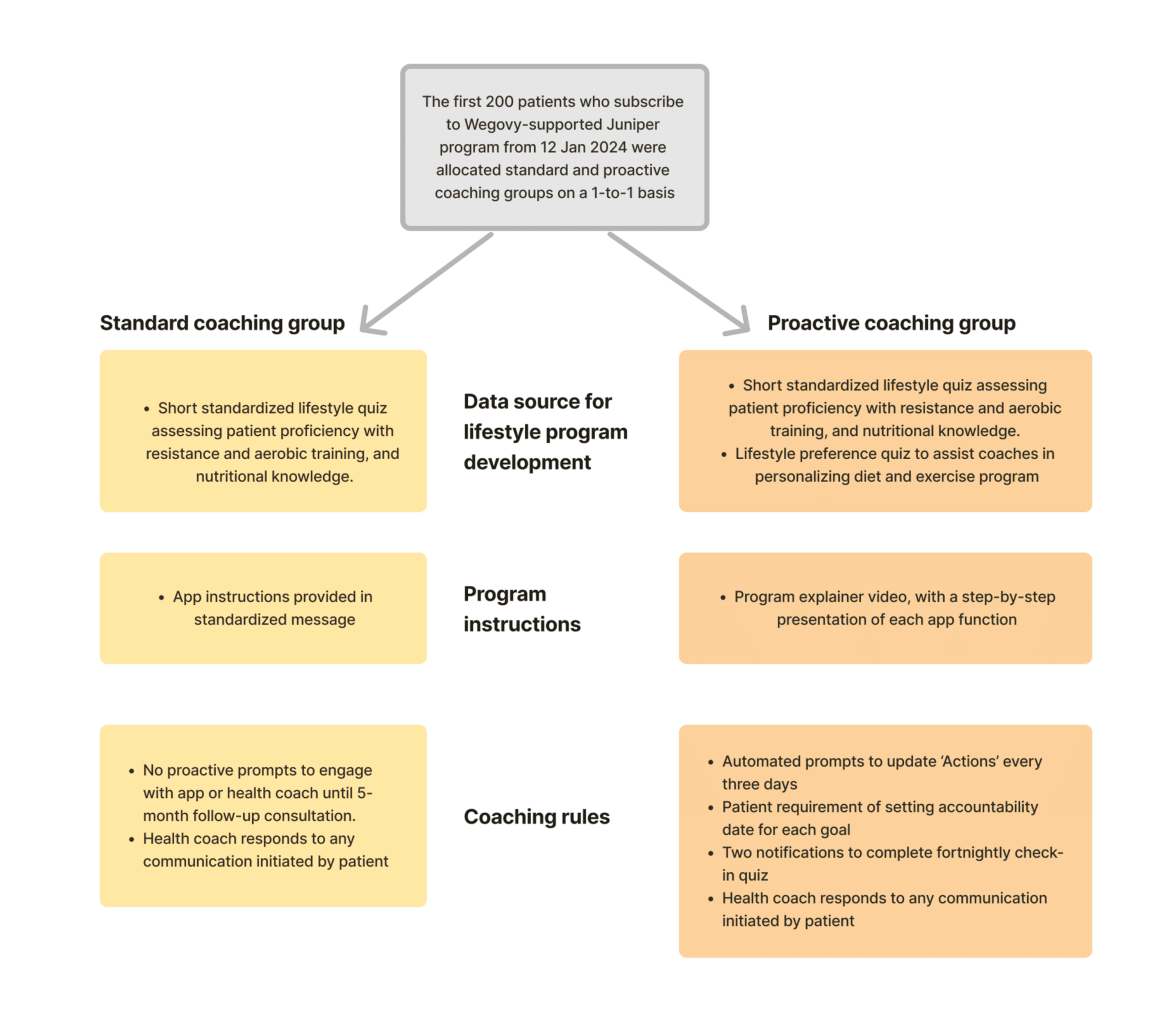
Flowchart comparison of two coaching groups

To maintain objectivity, patients who received proactive coaching were not informed they were part of an internal test, as Juniper UK reasonably assumed that the new coaching protocol would not deliver worse outcomes than the standard model. All patients consented to their de-identified data being used for research purposes. A visual comparison of the two coaching groups is presented in Figure 2.

Measures

Primary endpoints were the mean weight-loss percentage from baseline to 16-week follow-up and the mean number of patient messages to their MDT. The study’s secondary endpoints included the mean number of days when patients opened the program app, opened the goal tracker feature, and played a minimum of 80% of an educational video; and the proportion of patients who reached 5, 10 and 15 percent weight loss milestones. Data were only captured in the program app and goal tracker markers if patients had the respective pages open for a minimum of five seconds. To our knowledge, the engagement-related endpoints (mean number of messages; mean number of days opening the app, goal tracker and educational videos) have no precedent in existing obesity literature. They were selected in accordance with the WHO and NICE guidelines around continuous care in the hope of establishing informative foundational markers for future regulatory standards. 

Statistical analysis

All descriptive statistics were reported as means with standard deviations, along with frequency distributions (where relevant). For continuous-dependent variables such as weight loss and patient messages, two-sample t-tests were used to compare means between the two study groups. Chi-square tests were conducted to assess the correlation between the coaching group and the achievement of 5, 10 and 15 percent weight-loss milestones (yes/no). Pearson correlation tests were used to assess the association between continuous predictor and dependent variables, such as age and number of days when the program app was opened. All statistical analyses and visualizations were conducted on RStudio (version 2023.06.1+524).

## Results

One-hundred and fifty-four (77%) of the 200 study patients satisfied the study’s inclusion criterion, consisting of 70 patients from the proactive coaching group and 84 from the reactive group. Of the 44 patients who were excluded, 32 discontinued the program before week-16 follow-up and 12 failed to submit weight data within 100 and 120 days of program commencement. The mean age of the final cohort was 43.03 (±10.7) and the mean BMI was 33.87 (±6.12) kg/m^2^ (Table 1). Most patients were female (90.26%) and of Caucasian ethnicity (82.47%). Patients submitted follow-up weight data at a mean of 112.88 (±5.22) days after program initiation.

**Table 1:** Baseline characteristics

Demographic information	
Age, Mean±SD	43.03 (±10.7) years
Gender, n (%)	
Female	139 (91.57)
Male	44 (8.43)
Ethnicity, n (%)	
Caucasian	127 (82.47)
Asian including subcontinent	8 (5.19)
Black African of African Caribbean	6 (3.9)
Latino/Hispanic	6 (3.9)
Rather not say	5 (3.25)
Middle Eastern	2 (1.3)
Clinical information, Mean±SD	
BMI	33.87 (±6.12) kg/m^2^
Weight	94.93 (±19.41) kg

**Figure 3:**
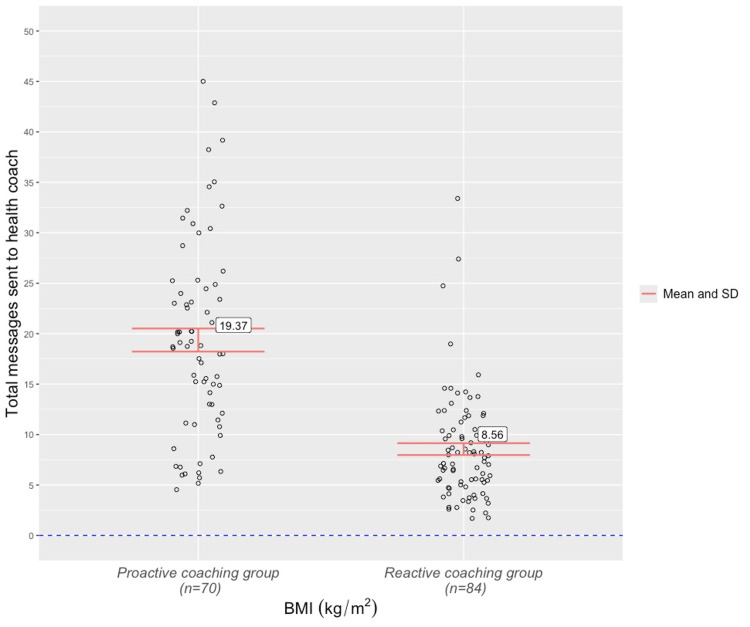
Number of patient messages to their health coach by the coaching group

The mean weight-loss percentage from baseline to week-16 follow-up was 9.47(±5.72) across the entire cohort. Patients in the proactive coaching group recorded a higher mean weight loss percentage (10.09 (±4.42)) than those from the reactive coaching group (8.96 (±6.6)), but a two-sample t-test revealed that this difference was not statistically significant, t(152) = -1.22, p = .22. For the entire cohort, the mean number of patient-to-coach messages over the 16-week study period was 13.05 (±9.03). A two-sample t-test found that proactive coaching patients sent a statistically higher number of messages to their health coaches (19.37(±9.58)) than reactive group patients (8.55(±5.39)), t(152) = 8.81, p <0.001 (Figure 3). 

A statistically significant difference was observed between the two groups in the number of days in which they opened the Juniper app, t(152) = -2.72, p <0.001. Whereas the proactive coaching group opened the app at a mean of 49.31 (±21.9) days throughout the study period, the reactive group recorded a mean of 40.06 (±20.3) days. Although a higher mean was observed in the proactive coaching group for the number of days opening the "Action" tracker (32.99 vs 28.5) and the number of days opening educational videos (5.07 vs 4.1), t-tests found that these differences were not statistically significant (Action tracker: t(152) = -1.43, p = 0.15; videos: t(152) = -1.89, p = 0.06).

Chi-square tests detected no statistical differences between the two groups in the proportion of patients who reached the 5 (X2(1, N = 154 = 1.68, p = 0.19)), 10 (X2(1, N = 154 = 0.07, p = 0.79)), or 15 (X2(1, N = 154 = 0.06, p = 0.8)) percent weight-loss milestones (Table 2). Across the full cohort, 84.44 % of patients lost a clinically meaningful amount of weight (≥5%), 47.4% lost at least 10 percent of their baseline weight, and 14.93% lost at least 15 percent.

**Table 2:** Chi-square contingency table of weight-loss milestones

	Proactive group (n=70)		Reactive group (n=84)				
	N	%	N	%	X^2^		p-value
>= 5% weight lost					1.68		0.19
Yes	62	88.6	68	81.0			
No	8	11.4	16	19.0			
>= 10% weight lost					0.07		0.79
Yes	34	48.6	39	46.4			
No	36	51.4	33	53.6			
>= 15% weight lost					0.06		0.8
Yes	11	15.7	12	14.3			
No	69	84.3	72	85.7			

Pearson tests found that age did not correlate with the number of patient messages (r(152) = -0.91, p = 0.36), weight loss (r(152) = -1.57, p = 0.12), or app opening frequency (r(152) = -0.05, p = 0.95). Initial BMI was also observed to have no significant effect on patient messages (r(152) = 0.14, p = 0.89), weight loss (r(152) = 0.27, p = 0.79), or app opening frequency (r(152) = -1.06, p = 0.29). Given the low number of non-Caucasian ethnic groups in the sample, a binary ethnicity variable was created (Caucasian vs non-Caucasian). Two-sample t-tests revealed that ethnicity did not significantly correlate with patient messages (t(152) = -1.08, p = 0.28), weight loss (t(152) = 0.98, p = 0.32), or app opening frequency (t(152) = 0.45, p = 0.65). Patient gender was found to be associated with weight loss (t(152) = 1.89, p = 0.04), but not the number of patient messages (t(152) = 0.11, p = 0.9) or days opening the Juniper app (t(152) = 0.95, p = 0.34). Whereas female patients lost a mean of 9.76 (±5.74) percent of their baseline weight, the mean weight-loss percentage among male patients was 6.88 (±5.06).

In the proactive group, 64.3 percent of patients reported at least one side effect compared to 62.9 percent in the reactive group, with a chi-square test revealing that this difference was not statistically significant (X2(1, N = 154 = 0.03, p = 0.85)) (Table 3). Of all reported side effects, only 2.9% and 3.6% were considered severe in the proactive and reactive groups, respectively. The two most common types of side effects in both groups were gastrointestinal issues and headaches. 

**Table 3:** Side effect incidence by type and severity p-values calculated via chi-square tests

	Proactive coaching group (n=70)	Reactive coaching group (n=84)	p-value
Side effect type – no (% of cohort)			
Gastrointestinal issues	37 (52.8)	51 (60.71)	0.327
Headaches	27 (38.5)	44 (44.04)	0.087
Fatigue or dizziness	14 (20)	12 (14.28)	0.346
Other	3 (4.29)	9 (10.7)	0.869
At least one side effect	44 (62.9)	54 (64.3)	0.854
Side effect severity – no. (% total side effects)			
Mild side effects	34(48.6)	42 (50)	0.860
Moderate side effects	18 (25.7)	22 (26.2)	0.947
Severe side effects	2 (2.9)	3 (3.6)	0.803

## Discussion

To the knowledge of the authors, this was the first study to measure the impact of lifestyle coaching design on engagement and effectiveness in a GLP-1 RA-supported DWLS. Previous research found that such services can improve access to obesity care relative to in-person services [7] and that patients tend to prefer the lifestyle coaching component of GLP-1 RA-supported DWLSs to be more proactive and personalized rather than relying on automated and/or patient-led prompts [31]. However, no studies have investigated whether this preference translated to improved patient outcomes or engagement. Moreover, neither of the two previous real-world studies of semaglutide treatment on non-diabetic weight-loss cohorts reported any patient-clinician engagement measures as neither intervention appeared to follow WHO and NICE guidance of using semaglutide as an adjunct to continuous lifestyle therapy [32,33]. 

The analysis discovered that patients of the Juniper UK DWLS who received proactive coaching sent a statistically higher number of messages to their health coach over the 16-week study period than patients whose coaching was reactive (19.37 vs 8.55). These mean figures convert to an average of 1.2 messages per week for the proactive group and 0.53 messages per week for the reactive group. Although messaging frequency guidelines are yet to be established in obesity or chronic care settings, the observed message frequency rate in the proactive group appears satisfactory from a care continuity perspective. Moreover, the rates observed in the two arms of this study lay a foundation for ongoing research into engagement with GLP-1 RA-supported DWLSs. Proactive coaching patients also opened the Juniper app on a significantly higher number of days than reactive coaching patients (49.31 vs 40.06 days). However, this app use disparity was considerably smaller (t = -2.72) than the one found in messaging frequency (t = -8.81) and may be of limited real-world significance. It is feasible that various patients opened the app without deriving any meaningful engagement benefits, such as thinking about exercise or recommended meals. It is also possible that patients in the proactive coaching group had higher latent motivation levels than those from the reactive group. Although no statistical differences were observed in the other engagement markers, the observed cohort-wide rate of opening the Action tracker (30.54 days over the study period) was arguably significant in itself, as it indicated that Juniper patients engaged with goal-specific content every 3.7 days. Again, no existing data is available for comparison, but the engagement rate would feasibly satisfy future standards of obesity care continuity.

The analysis also found no statistically significant difference in the weight-loss outcomes of the reactive and proactive coaching groups. There are several possible explanations for this finding. Firstly, this outcome may reflect the diversity of personalities in modern society, with some patients preferring high engagement with clinicians and others responding just as well to asynchronous material without feeling inclined to return messages or ask further questions. Patients with a preference for high engagement who were allocated to the reactive group still had the option of messaging their health coach as often as they liked, and Figure 3 showed that several of this group’s patients recorded a frequency of messages comparable to the proactive group mean. Secondly, the discovery could indicate the insignificance of coaching intensity relative to program design. It may be that a patient’s awareness that they can access multidisciplinary support whenever necessary is more important than MDT messaging frequency when a program contains clear instructional material and a well-designed program app and accountability tool (such as the Juniper ‘Action’ tracker). Both groups opened the app and ‘Action’ tracker regularly (roughly 2-3 times per week) and watched over 80 percent of an educational video between 4 to 5 times over the study period. It is possible that these markers are a more reliable reflection of patient engagement than MDT-messaging frequency among various patients, and perhaps even more reliable after a certain number of messages have been exchanged. Finally, although the difference in mean weight loss between the two groups was not statistically significant, some observers may consider the disparity between 10.1% (proactive group) and 8.9% (reactive group) to be clinically meaningful. It is possible that a correlation between weight loss and messaging frequency would have manifested over a longer study period. However, running long-term comparative analyses of real-world semaglutide-supported services remains a challenge for unsubsidized providers given the high cost of the medication. 

Even though no statistical correlation was observed between coaching style and weight loss, the mean 16-week weight-loss percentage of 10.1(±4.42) observed in the proactive group represents a significant discovery in itself. The two previous real-world semaglutide obesity studies reported a mean three-month weight loss of 6.3 and 6.6%, respectively [32,33]. Although 16 weeks equate to 3.68 months (three weeks longer than three months), mean weight loss of 10.1% at this point can be reasonably interpreted as superior to 6.3% and 6.6% at three months. An arguably more significant disparity was in the proportion of patients who achieved clinically meaningful (≥5%) weight loss relative to the 2 F2F interventions. Whereas 20% and 53.7% of the Athens endocrine and Mayo Clinic cohorts reached this milestone at three months, 88.6% from the Juniper UK proactive coaching group did the same at 3.68 months. It is likely that these weight-loss disparities reflect the additional benefits weight-loss patients derive from combining their semaglutide treatment with continuous multidisciplinary lifestyle therapy. Whereas patients in the two previous real-world semaglutide studies appeared to receive minimal lifestyle advice, diet and exercise coaching is the centrepiece of the Juniper UK program. Thus, these findings offer some early validation of the WHO and NICE guidance for GLP-1 RA-supported obesity interventions [17,18]. However, it needs to be stressed that this study’s failure to run for its intended 6-month duration is a significant limitation. It remains possible that the Juniper program’s 6-month weight-loss outcomes would have been comparable to those of the previous 2 F2F interventions. Moreover, while the study’s drop-out rate (23%) is lower than the percentage of patients who discontinued the Athens (42%) and Mayo Clinic (44.2%) studies between months 3 and 6, these figures cannot be reasonably compared given that neither of the latter two studies provided data on program initiation numbers (i.e., the number of patients who started treatment and were excluded before month 3). It is likely that a significant proportion of Juniper patients would have discontinued the study between week 16 and its intended six-month endpoint given that the program’s already high cost would have been at its most expensive during that period (£299). Side effect incidence was very similar in the Juniper (63.6%) and Mayo Clinic cohorts (65%), whose rate was higher than that reported in the Athens-based study (48.5%). Finally, the finding that female patients lost significantly more weight than male patients across the full cohort (9.76% vs 6.88%) is notable. While previous studies of the Juniper DWLS have not detected the same trend [27,29,35], none of those studies nor the current one have contained a high percentage of male patients. A possible explanation of the gender discrepancy in this study is a higher health literacy among female patients [36], which may have manifested in more determination to commit to the program and improve their health. Ultimately, the study’s gender discovery highlights the need for a focused investigation of male DWLS patients. 

In addition to a dedicated gender analysis of DWLS outcomes, researchers should consider conducting comparable analyses of other GLP-1 RA-supported DWLSs. Researchers are also encouraged to perform analyses over longer periods and with a medication-only control group. Such studies will likely remain financially challenging for service providers until GLP-1 RA medications become more affordable and/or government or industry subsidies are offered.

This study’s strengths included its novelty, its non-interference with patient experience in the Juniper program, and its collection of multiple engagement data for all study subjects. The study also contained several limitations. Firstly, the study duration was cut from 26 weeks down to 16 weeks due to Juniper’s budget constraints. Although the study still generated multiple significant outcomes, further important discoveries may have been made had the study continued for its intended duration. As discussed above, a 26-week investigation would have likely generated a higher drop-out rate given the Juniper program’s high costs, which peak from week 17 onwards. It is also possible that a significant number of patients would have discontinued between weeks 16 and 26 due to medication supply issues, dissatisfaction with results or the Juniper service, or changes in life circumstances, as previous adherence studies have reported [28,30]. Following this, the Juniper DWLS’ high initial cost represented a second study limitation, as it would have likely excluded people from lower socioeconomic groups. Thirdly, the sample included a disproportionately high percentage of females and Caucasians and therefore was not representative of the diverse British population. Although this study and previous investigations have not detected significant ethnic group differences in weight-loss outcomes of semaglutide-supported interventions, this may be due to the underrepresentation of non-Caucasian subjects in their samples. Future studies should endeavor to include more diverse cohorts so that more generalizable conclusions can be drawn. Fourthly, weight data within the study period (up to 120 days) were missing from patients who failed to satisfy the data entry inclusion criterion and could not be reported. Finally, investigators could not account for the reasons behind the decision of 44 patients to discontinue the program before study completion. Previous adherence studies of the Juniper program have indicated that GLP-1 RA supply issues, program cost, dissatisfaction with results, and intolerable side effects are among the most common reasons for early discontinuation [28,30].

## Conclusions

Previous research found that semaglutide-supported DWLSs can be effective in treating non-diabetic patients with overweight and obesity and that patients tend to prefer the lifestyle coaching component of these services to be proactive and personalized rather than reactive and standardized. However, no previous study had assessed the degree to which these different coaching methods affect patient engagement and effectiveness in a non-subsidized DWLS that supplements coaching with semaglutide. The findings from this study indicate that app and lifestyle program design may be more important than coaching intensity in delivering continuous obesity care through digital platforms. Moreover, the discovery that the Juniper cohort’s 16-week weight-loss outcomes were significantly better than the three-month outcomes of real-world semaglutide interventions with minimal lifestyle input gives foundational support to WHO and NICE recommendations. Real-world DWLSs should endeavour to collect and publish data on the engagement measures used in this study to facilitate the establishment of industry-wide regulatory standards. Research of longer duration is needed to deliver further conclusions about the design and effectiveness of semaglutide-supported DWLSs.

## References

[1] (2024). Prevalence of Obesity. https://www.worldobesity.org/about/about-obesity/prevalence-of-obesity.

[2] Anekwe CV, Jarrell AR, Townsend MJ, Gaudier GI, Hiserodt JM, Stanford FC (2020). Socioeconomics of obesity. Curr Obes Rep.

[3] Javed Z, Valero-Elizondo J, Maqsood MH (2022). Social determinants of health and obesity: findings from a national study of US adults. Obesity (Silver Spring).

[4] Verde L, Frias-Toral E, Cardenas D (2023). Editorial: environmental factors implicated in obesity. Front Nutr.

[5] Hall KD, Kahan S (2018). Maintenance of lost weight and long-term management of obesity. Med Clin North Am.

[6] Finucane FM, Gibson I, Hughes R (2023). Factors associated with weight loss and health gains in a structured lifestyle modification programme for adults with severe obesity: a prospective cohort study. Front Endocrinol (Lausanne).

[7] Talay L, Vickers M, Loftus S (2024). Why people with overweight and obesity are seeking care through digital obesity services: a qualitative analysis of patients from Australia’s largest digital obesity provider. Telemed Rep.

[8] Pi-Sunyer X, Astrup A, Fujioka K (2015). A randomized controlled trial of 3.0mg of Liraglutide in weight management. N Engl J Med.

[9] Wilding JP, Batterham RL, Calanna S (2021). Once-weekly semaglutide in adults with overweight or obesity. N Engl J Med.

[10] Davies M, Faerch L, Jeppesen O (2021). Semaglutide 2.4mg once a week in adults with overweight or obesity, and type 2 diabetes (Step 2): a randomized, double-blind, double-dummy, placebo-controlled, phase 3 trial. Lancet.

[11] Wadden TA, Bailey TS, Billings LK (2021). Effect of subcutaneous semaglutide vs placebo as an adjunct to intensive behavioral therapy on body weight in adults with overweight or obesity: The STEP 3 Randomized Clinical Trial. JAMA.

[12] Bessesen DH, Van Gaal LF (2018). Progress and challenges in anti-obesity pharmacotherapy. Lancet Diabetes Endocrinol.

[13] Kim MS (2022). The neural basis of weight control and obesity. Exp Mol Med.

[14] Ard J, Fitch A, Fruh S, Herman L (2021). Weight loss and maintenance related to the mechanism of action of glucagon-like peptide 1 receptor agonists. Adv Ther.

[15] Tullman Tullman, G G (2024). Weight Loss Drugs are Not the Only Answer: Why We Must Redefine the Obesity Journey. Why we must redefine the obesity journey, Jan 10.

[16] Dowsett G, Yeo G (2023). Are GLP-1R agonists the long-sought-after panacea for obesity?. Trends Mol Med.

[17] (2023). Health Service Delivery Framework for Prevention and Management of Obesity. Geneva.

[18] (2023). Semaglutide for Managing Overweight and Obesity. Semaglutide for managing overweight and obesity Sep.

[19] (2024). Appointments in General Practice. November.

[20] Crompvoets PI, Nieboer AP, van Rossum EF, Cramm JM (2024). Perceived weight stigma in healthcare settings among adults living with obesity: a cross-sectional investigation of the relationship with patient characteristics and person-centred care. Health Expect.

[21] Prior SJ, Luccisano SP, Kilpatrick ML, Murfet GO (2022). Assessment and management of obesity and self-maintenance (AMOS): an evaluation of a rural, regional multidisciplinary program. Int J Environ Res Public Health.

[22] Hill JL, You W, Zoellner JM (2014). Disparities in obesity among rural and urban residents in a health disparate region. BMC Public Health.

[23] Golovaty I, Hagan S (2024). Direct-to-consumer platforms for new antiobesity medications - concerns and potential opportunities. N Engl J Med.

[24] Hinchliffe N, Capehorn MS, Bewick M, Feenie J (2022). The potential role of digital health in obesity care. Adv Ther.

[25] (2019). Accenture 2019 Digital Health Consumer Survey. Accenture.

[26] Payne Payne, H H (2024). No Doctor, No Script, No Worries: Here’s Your Ozempic. Medical Republic, 10 May.

[27] Talay L, Alvi O (2024). Digital healthcare solutions to better achieve the weight loss outcomes expected by payors and patients. Diabetes Obes Metab.

[28] Talay L, Vickers M (2024). Patient adherence to a real-world digital, asynchronous weight program in Australia That combines behavioural and GLP-1 RA therapy: a mixed methods study. Behav Sci (Basel).

[29] Talay L, Vickers M (2024). Effectiveness and care continuity in an app-based, glucagon-like peptide-1 receptor agonist-supported weight-loss service for women with overweight and obesity in the UK: a real-world retrospective cohort analysis. Diabetes Obes Metab.

[30] Talay L, Vickers M (2024). Patient adherence to a digital real-world GLP-1 RA-supported weight-loss program in the UK: a retrospective cohort study. J Community Med Public Health.

[31] Talay L, Vickers M, Wu S (2024). Patient satisfaction with an Australian digital weight-loss service: a comparative retrospective analysis. Telemed Rep.

[32] Ghusn W, De la Rosa A, Sacoto D (2022). Weight loss outcomes associated with semaglutide treatment for patients with overweight or obesity. JAMA Netw Open.

[33] Tzoulis P, Batavanis M, Baldeweg S (2024). A real-world study of the effectiveness and safety of semaglutide for weight loss. Cureus.

[34] Novo Nordisk. (2024). Wegovy: Semaglutide Injection 2.4mg. Wegovy: Semaglutide Injection 2.4mg. Novo Nordisk Inc, 2024.Bagsvaerd, Denmark.

[35] Talay L, Vickers M, Ruiz L (2024). Effectiveness of an email-based, semaglutide-supported weight-loss service for people with overweight and obesity in Germany: a real-world retrospective cohort analysis. Obesities.

[36] Ayotte BJ, Allaire JC, Bosworth H (2009). The associations of patient demographic characteristics and health information recall: the mediating role of health literacy. Neuropsychol Dev Cogn B Aging Neuropsychol Cogn.

